# Frequent acquisition of simian foamy viruses from gorillas, chimpanzees and monkeys through severe bites in central African hunters with no evidence for intra-familial dissemination

**DOI:** 10.1186/1742-4690-8-S1-A237

**Published:** 2011-06-06

**Authors:** Edouard Betsem, Tortevoye Patricia, Froment Alain, Antoine Gessain

**Affiliations:** 1 EPVO unit, Department of Virology, Institut Pasteur, Paris, France; 2 Faculté de Médecine et des Sciences Biomédicales, Université de Yaoundé 1, Cameroun; 3 IRD, Musée de l’Homme, Paris, France

## Background

Human infection by Simian Foamy Virus (SFV) can be acquired mainly through bites in persons occupationally exposed to non-human primates (NHP) or in natural settings.

This study aimed at getting better knowledge on the risk factors associated to presence of SFV infection in humans at risk for such zoonosis and, searching for intra-familial dissemination from the original infected cases.

## Results

We studied 1257 people from the general adult population (mean age 53 yrs, women 600 and men 657) and 182 individuals, mostly men, who all encountered a NHP with a resulting bite or scratch. All of these, either Baka Pygmies (380) or Bantou (1020) people live in villages in South Cameroon, a rainforest natural habitat for several NHP species. A specific SFV Western blot was used for plasma and two nested PCR (integrase and LTR) were done on all the positive/borderline samples by serology. In the general population, 2/1257 persons were found SFV infected, one by a gorilla and one by a monkey FV. In the second group, 35/182 (19.2%) persons were SFV positive. They were mostly infected (31/35) by apes FV (mainly gorilla), while infection by monkey FV was less frequent (3/35). Of the 30 wives and 17 children from families of FV positive persons, only one plasma, from a wife was WB positive.

## Conclusion

We demonstrate a high level of transmission of SFVs into humans in natural settings specifically following severe apes and monkey bites and a viral persistence over several years. Secondary transmissions remain in question.

